# An Immunomodulating Fatty Acid Analogue Targeting Mitochondria Exerts Anti-Atherosclerotic Effect beyond Plasma Cholesterol-Lowering Activity in apoE^-/-^ Mice 

**DOI:** 10.1371/journal.pone.0081963

**Published:** 2013-12-04

**Authors:** Rita Vik, Marco Busnelli, Cinzia Parolini, Bodil Bjørndal, Sverre Holm, Pavol Bohov, Bente Halvorsen, Trond Brattelid, Stefano Manzini, Giulia S. Ganzetti, Federica Dellera, Ottar K. Nygård, Pål Aukrust, Cesare R. Sirtori, Giulia Chiesa, Rolf K. Berge

**Affiliations:** 1 Department of Clinical Science, University of Bergen, Bergen, Norway; 2 Department of Pharmacological and Biomolecular Sciences, Università degli Studi di Milano, Milan, Italy; 3 Research Institute of Internal Medicine, Oslo University Hospital Rikshospitalet, University of Oslo, Oslo, Norway; 4 Institute of Clinical Medicine, University of Oslo, Oslo, Norway; 5 National Institute of Nutrition and Seafood Research, NIFES, Bergen, Norway; 6 Department of Heart Disease, Haukeland University Hospital, Bergen, Norway; 7 Section of Clinical Immunology and Infectious Diseases, Oslo University Hospital Rikshospitalet, University of Oslo, Oslo, Norway; 8 K.G. Jebsen Inflammation Research Centre, University of Oslo, Oslo, Norway; St. Joseph's Hospital and Medical Center, United States of America

## Abstract

Tetradecylthioacetic acid (TTA) is a hypolipidemic antioxidant with immunomodulating properties involving activation of peroxisome proliferator-activated receptors (PPARs) and proliferation of mitochondria. This study aimed to penetrate the effect of TTA on the development of atherosclerotic lesions in apolipoprotein (apo)-E^-/-^ mice fed a high-fat diet containing 0.3% TTA for 12 weeks. These mice displayed a significantly less atherosclerotic development vs control. Plasma cholesterol was increased by TTA administration and triacylglycerol (TAG) levels in plasma and liver were decreased by TTA supplementation, the latter, probably due to increased mitochondrial fatty acid oxidation and reduced lipogenesis. TTA administration also changed the fatty acid composition in the heart, and the amount of arachidonic acid (ARA) and eicosapentaenoic acid (EPA) was reduced and increased, respectively. The heart mRNA expression of inducible nitric oxidase (NOS)-2 was decreased in TTA-treated mice, whereas the mRNA level of catalase was increased. Finally, reduced plasma levels of inflammatory mediators as IL-1α, IL-6, IL-17, TNF-α and IFN-γ were detected in TTA-treated mice. These data show that TTA reduces atherosclerosis in apoE^-/-^ mice and modulates risk factors related to atherosclerotic disorders. TTA probably acts at both systemic and vascular levels in a manner independent of changes in plasma cholesterol, and triggers TAG catabolism through improved mitochondrial function.

## Introduction

Atherosclerosis is a complex vascular disease with a bidirectional interaction between lipids and inflammation as a major feature. These interactions involve monocytes/macrophages, T cells, vascular smooth muscle cell (SMC) and endothelial cells, and in addition to inflammation and lipid deposition, matrix remodelling is an important characteristic of the atherosclerotic lesion [1]. The liver, which is a central regulator of fatty acid and glucose metabolism, is also involved in an atherosclerosis development and systemic low-grade inflammation characterizing this disorder, as demonstrated by the increased subclinical atherosclerosis in patients with non-alcoholic fatty liver disease (NAFLD) [2]. Additionally, there is growing evidence supporting a role of mitochondrial dysfunction that also involves enhanced oxidative stress in the pathogenesis of atherosclerosis [3,4]. Despite state-of-the-art cardiovascular treatment including the use of statins, 38% of all deaths in North America is related to atherosclerosis, and it is the most common cause of death in European men over 45 and in woman over 65 years of age [5]. The growing epidemic of obesity and diabetes associated with a metabolic phenotype characterized by dyslipidemia may further contribute to an increased risk of cardiovascular disease. These factors force us to consider new strategies for prevention and treatment of atherosclerotic disorders. Although statins have been shown to possess anti-inflammatory properties, modulation of the non-resolving inflammation that characterizes the atherosclerotic process is still a therapeutic challenge. 

Tetradecylthioacetic acid (TTA) is a modified saturated fatty acid (SFA) analogue made of 16 carbons, plus a sulphur atom inserted in the 3-position from the carboxylic end. Following cellular uptake, TTA is converted to TTA-CoA [6], which is a good substrate for the carnitine palmitoyltransferase system (CPT1 and CPT2) [7], transporting acyl-CoA into the mitochondria. However, due to the sulphur atom at the 3 position TTA is blocked for β-oxidation [8], and the S-substitution also contribute to the antioxidant effect [9]. TTA stimulates mitochondrial fatty acid oxidation of normal β-oxidizable fatty acids [10] and induces mitochondrial proliferation in rat hepatocytes [11]. As a pan-ligand for members of the peroxisome proliferator-activated receptor (PPAR) family of nuclear hormone receptors, TTA regulates expression of fatty acid metabolizing enzymes, in particular those of the catabolic pathway [12–14]. TTA is reported to have both cholesterol- and triacylglycerol (TAG) lowering effect in in psoriasis patients [8]. The increased mitochondrial fatty acid oxidation and mitochondrial proliferation seem to be important in regulating plasma TAG level [11,12]. Moreover, TTA is an antioxidant [15] with immunomodulating properties, able to lower plasma level of tumor necrosis factor (TNF)-α, vascular cell adhesion molecule (VCAM)-1 and interleukin (IL)-8 *in vitro* [16] as well as *in vivo* [17]. The anti-inflammatory effects of TTA appear to involve both PPARα-dependent and –independent pathways [16]. Finally, TTA is found to be present in the coronary wall [18], suggesting a potential for a direct effect on coronary atherosclerosis.

Based on its hypolipidemic properties as well as its anti-oxidative and anti-inflammatory potential involving mitochondrial related mechanisms, we examined the ability of TTA to modulate atherosclerotic lesion development through an intervention study in apolipoprotein E-deficient (apoE^-/-^) mice. ApoE^-/-^ mice are a widely recognized model for hypercholesterolemia that spontaneously develops atherosclerotic lesions, even on a standard chow diet with low fat and no cholesterol [19] and have been used to study the effect of pharmacological or dietary treatments on atherosclerosis development [20]. 

## Materials and Methods

### Animals and diets

 The animal study was conducted according to the national (D.L. 116, G.U. Suppl. 40, February 18, 1992, Circolare No. 8, G.U July 1994) and international laws and policies (EEC Council Directive 86/609, OJL 358, 1, December 12, 1987: Guide for the Care and Use of Laboratory Animals, United States National Research Council, 1996). The Italian Ministry of Health approved the protocol. Mice were kept under standard laboratory conditions with temperature 22±1°C, dark/light cycles of 12/12 h, relative humidity 55±5% and 20 air changes per hour. Food and tap water was given *ad libitum*.

Twenty-four female apoE ^-/-^ mice (North Carolina, Charles River Laboratories, Italy), 8 weeks old, were randomly divided, based on body weight, into two groups of 12 animals. Mean cholesterol levels pre-treatment were not different between groups (8.16±0.80 vs 7.73±0.62 mmol/L, p=0.429). The control group was fed a high-fat diet (23.7% w/w) consisting of 21.3% lard (a generous gift from Ten Kate Vetten BV, Musselkanaal, Netherlands) and 2.4% soy oil (Dyets. Inc., Betlehem, PA, USA), with 25% w/w casein (Dyets Inc., Bethlehem, PA, USA) as protein source, while the intervention diet was supplemented with 0.3% w/w TTA, and TTA was synthesized as previously described [21]. The energy contribution from fat, carbohydrates, and protein were 46.9%, 32.8% and 20.3%, respectively and the full composition of the diets is given in Table **S1**. Mice had free access to tap water and feed during the 12 weeks of feeding.

### Blood and tissue harvesting

Day 0 and after 28, 56 and 77 days of dietary treatment, blood was collected from the retro-orbital plexus into tubes containing 0.1% (w/v) EDTA after an overnight fasting. Blood samples were chilled on ice for at least 15 minutes, centrifuged and stored at -80°C prior to analysis. After 84 days of treatment, mice were anesthetized with 2% isoflurane (Forane, from Abbot Laboratories Ltd, Illinois, USA) and blood was removed by perfusion with phosphate-buffered saline (PBS). Aorta was rapidly dissected from the aortic root to the iliac bifurcation, periadventitial fat was removed and aorta was pinned flat on a black wax surface in ice-cold PBS and photographed. After photographing, aorta was immediately put in a tissue-freezing medium, snap-frozen in liquid nitrogen and stored at -80°C. For histological/immunohistochemical analysis, six hearts from each group were removed, fixed in 10% formalin for 30 min and transferred into PBS containing 20% sucrose (w/v) overnight at 4°C before being embedded in OCT compound (Sakura Finetek Euope B.V., Alphen aan den Rijn, The Netherlands) and stored at -80°C. An equal subset of hearts was dedicated to qPCR analysis and immediately snap-frozen in liquid nitrogen. Livers from all animals were harvested, and a portion was embedded in OCT compound. The embedded portion and the remaining tissue were snap-frozen and stored at -80°C until further processing. 

### En face analysis

Images of the aorta were captured with a stereomicroscope-dedicated camera (IC80 HD camera, MZ6 microscope, Leica Microsystems, Germany), and analyzed with ImageJ image processing program (http://rsb.info.nih.gov/ij/). An operator blinded to dietary treatment quantified atherosclerosis. 

### Aortic sinus and liver histology/immunohistochemistry

Serial cryosections (7 μm thick) of the aortic sinus were cut and stained with hematoxylin and eosin (Bio-Optica, Milano, Italy) to evaluate plaque area and with Masson’s Trichrome (04-010802, Bio-Optica, Milano, Italy) to evaluate extracellular matrix deposition. Oil red O staining was used to detect intraplaque neutral lipids (Sigma-Aldrich, St. Louis, MO, USA). Seven μm thick sections were also cut from the liver and stained with Red Oil O. 

Macrophages and lymphocytes were detected using an anti-F4/80 antibody (ab6640, Abcam, Cambridge, UK), and an anti-CD3 antibody (ab16669, Abcam, Cambridge, UK), respectively. A biotinylated secondary antibody was used for streptavidine-biotin-complex peroxidase staining (Vectastain Abc Kit, Vector Laboratories, Peterborough, UK). 3,3’-Diaminobenzidine (DAB) was used as chromogen (Sigma-Aldrich, St. Louis, MO, USA), and sections were counterstained with hematoxylin (Gill’s Hematoxylin, Bio-Optica, Milano, Italy).

The Aperio ScanScope GL Slide Scanner (Aperio Technologies, Vista, CA, USA), equipped with a Nikon 20x/0.75 Plan Apochromat objective producing a 0.25 μm/pixel scanning resolution and the Aperio ImageScope software (version 8.2.5.1263), was used to acquire and process digital images with a 40x magnification. An operator blinded to dietary treatment quantified the plaque area, extracellular matrix and lipid composition, as well as inflammatory cell infiltrate.

### Plasma and liver lipids and fatty acid composition in heart and plasma

Liver lipids were extracted according to Bligh and Dyer [22], evaporated under nitrogen, and redissolved in isopropanol before analysis. Lipids from liver extracts or plasma were then measured enzymatically on a Hitachi 917 system (Roche Diagnostics GmbH, Mannheim, Germany) using the TAG (GPO-PAP), cholesterol (total, free, LDL and HDL) kit (CHOD-PAP) from Roche Diagnostics, phospholipid kit from bioMérieux SA (Marcy l'Etoile, France) and non-esterified fatty acid (NEFA) kit from DiaSys Diagnostic Systems GmbH (Holzheim, Germany). Total plasma fatty acid composition in heart and plasma was analyzed as described previously [23]. 

### Hepatic enzyme activities

Livers were homogenized and a post-nuclear fraction made as described earlier [24]. The activity of carnitine palmitoyltransferase (CPT)-1 was performed according to *Bremer* [25], but with some modifications: the reaction mix contained 17.5 mM HEPES pH 7.5, 52.5 mM KCl, 5 mM KCN, 100 mM palmitoyl-CoA, and 6.67 mg BSA/mL. 100 μM [methyl-14C]-L-carnitine (1100 cpm/ηmol) inititated the reaction, and 30 μg total protein was used. The assay conditions for CPT-2 were identical except that BSA and KCN was omitted and 0.01% Triton X-100 was included, and a total of 35 μg was used. 

Acyl-CoA oxidase (ACOX)-1 activity was measured using 20 μg protein, as described by *Madsen et al* [26].. 3-hydroxy-3-methylglutaryl coenzyme A synthase (HMG-CoA) were measured in the post-nuclear fraction as described by Skorve et al. [27], and citrate synthase activity was measured as previously described [28].

### Gene expression analysis

Total cellular RNA was extracted from liver, homogenates from the whole heart and from pooled aortic arch segments, and cDNA was produced as previously described [29]. Quantitative real-time PCR was performed on an ABI prisme 7900 H sequence detection system from Applied Biosystems with Sarstedt 384 well multiply-PCR Plates (Sarstedt Inc., Newton, NC, USA) using probes and primers from Applied Biosystems. The primers used are listed in Table **S2**. Six different reference genes were included for liver and heart, and three for aorta: 18s (Kit-FAM-TAMRA (Reference RT-CKFT-18s)) from Eurogentec, Belgium, ribosomal protein, large, P0 (*Rplp0*, Gene ID 11837), hypoxanthine guanine phosphoribosyl transferase 1 (*Hprt1*, AX-045271-00), ribosomal protein, large, 32 (*Rpl32*, AX-055111-00), polymerase II (DNA directed) polypeptide A, (*Polr2a*, AX-046005-00) and TATA-box binding protein (Tbp, AX-041188-00) from Thermo Fisher Scientific. GeNorm were used to evaluate the reference genes [30], and data normalized to *Rplp0* and *Rpl32* are presented for liver, *Hprt1* for heart and *18S*, *Rplp0* and *Hprt1* for aortic arch segment.

### Measurements of inflammatory markers

Levels of IL-1α, IL-1β, IL-6, IL-10, IL-17, interferon (IFN)-γ, monocyte chemoattractant protein (MCP)-1 and TNF-α were analyzed on plasma samples collected at day 77 after treatment by Multiplex suspension technology using a customized Bio-Plex Pro Mouse assay (Bio-Rad Laboratories, Hercules, CA). 

### Statistical analysis

Data sets were analyzed using Prism Software (GraphPad Prism version 5.0; GraphPad Prism, San Diego, CA, USA) to determine statistical significance. The results are shown as means of 3 to 12 animals per group with their standard deviations. Normal distribution was determined by the Kolmogorov-Smirnov test (with Dallal-Wilkinson-Lilliefors *P* value). Unpaired *t*-test was performed to evaluate statistical differences between two groups, or Mann Whitney test when values were not normally distributed. *P*-values <0.05 were considered significant.

## Results

### TTA effects on atherosclerosis development

**Figure 1:**
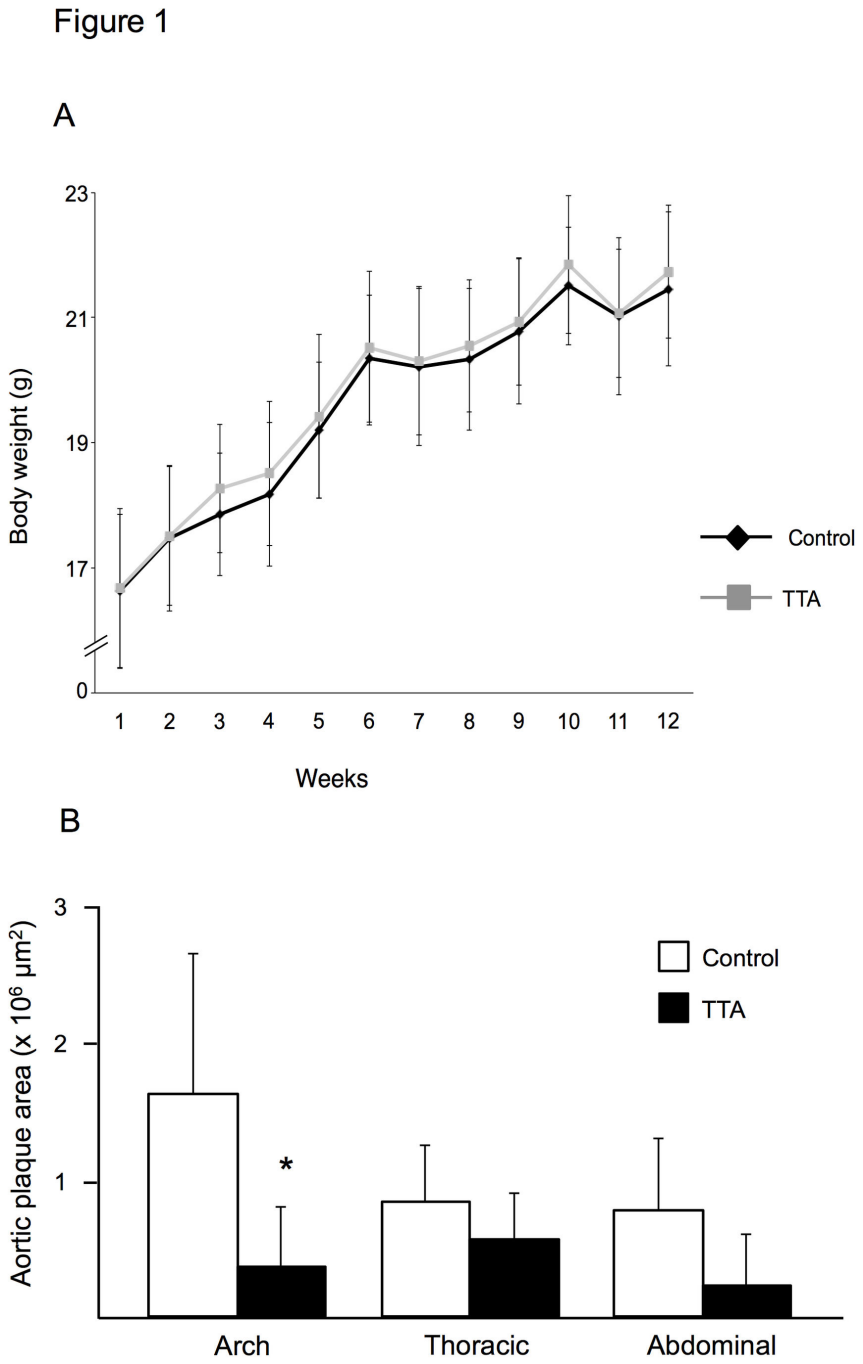
Effects of TTA on body weight and aortic atherosclerosis development apoE^-/-^ mice fed a high-fat diet (control) or a diet supplemented with 0.3% TTA. After 12 weeks of diet induction, TTA-fed mice displayed the same growth curve as controls (**A**). Whole aorta was collected and en-face analysis was performed to quantify aortic surface covered by atherosclerotic plaques (**B**). Data are shown as means ± SD for 6-12 mice for each diet. Unpaired *t*-test was used to detect statistical significance. *P<0.05 vs. control.

**Figure 2:**
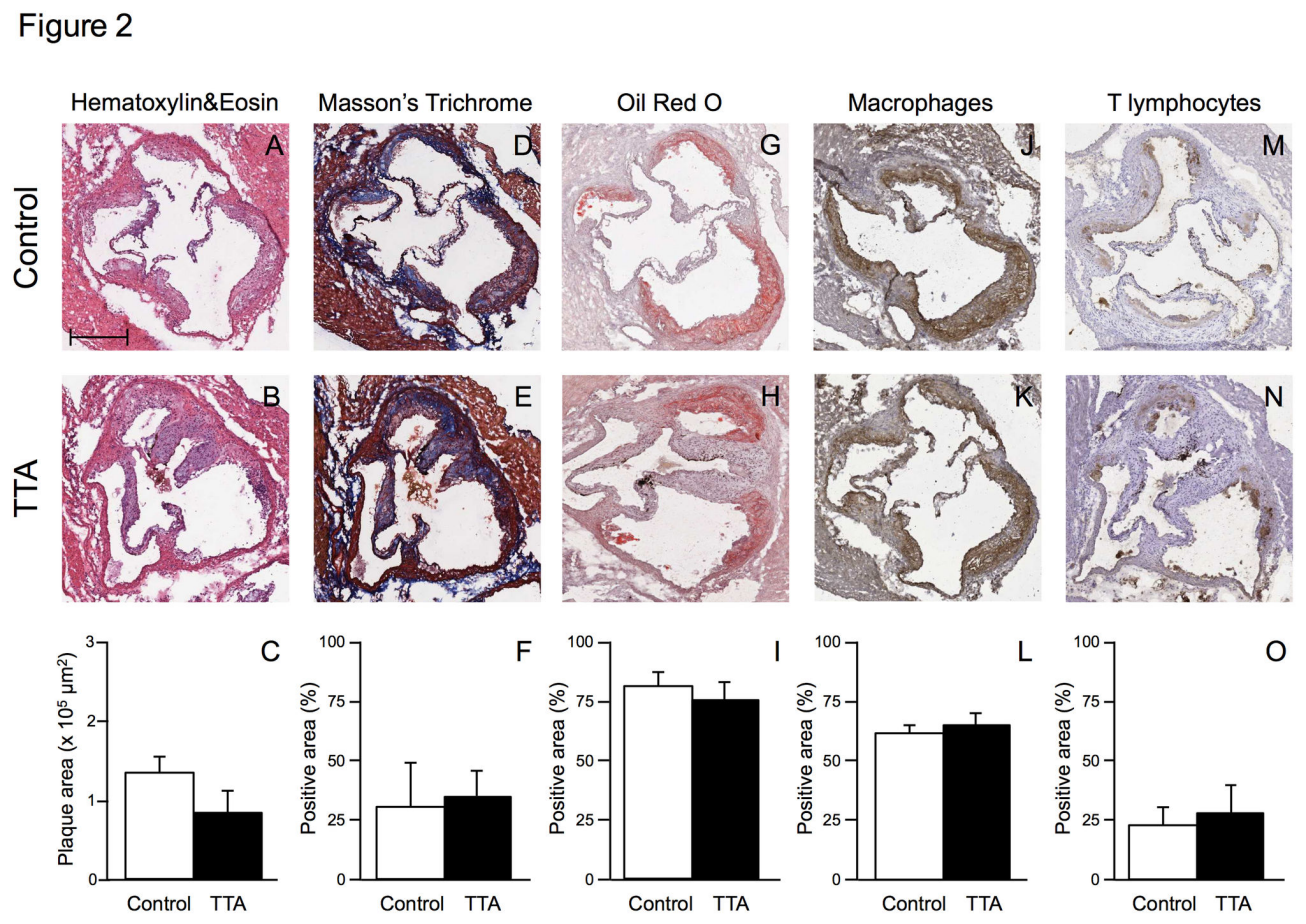
Histological and immunohistochemical characterization of plaques at the aortic sinus in apoE^-/-^ mice fed a high-fat diet (control) or a diet supplemented with 0.3% TTA for 12 weeks. Representative photomicrographs and quantification of maximum plaque area (panels A-C). Representative photomicrographs and quantification of extracellular matrix deposition (panels D-F), lipid deposition (panels G-I), macrophages (panels **J-L**) and T lymphocytes (panels M-O). Bar in panel A = 100 μm. Data are shown as means ± SD for 6 mice for each diet.

After treatment, mice fed TTA displayed the same body weight as controls (Figure **1A**). TTA diet administration significantly inhibited the development of atherosclerotic lesions at the aortic arch compared with the control diet (Figure **1B**). A lower atherosclerotic development was also observed in TTA-treated mice at the thoracic and abdominal aorta without reaching statistical significance (Figure **1B**). However, considering the entire aortic surface, TTA-treatment significantly inhibited atherosclerosis development (0.39 ± 0.22 x 10^6^ μm^2^ in TTA-mice vs 1.09 ± 0.50 x 10^6^ μm^2^ in controls; p=0.016, Figure **1B**). In addition, a tendency towards a decrease in lesion area was observed at the aortic sinus of TTA mice compared with control mice (1.49 ± 0.30 x 10^5^ μm^2^ vs. 2.02 ± 0.31 x 10^5^ μm^2^, respectively; p=0.068; Figure 2A-C). In contrast to the decrease in lesion size, the histological/immunohistochemical characterization of atherosclerotic lesions did not show any significant difference in plaque composition between controls and TTA mice, displaying a comparable percentage of extracellular matrix (30.31 ± 18.25 % vs. 29.75 ± 15.89 %; Figure 2D-F), lipids (79.68 ± 6.45 % vs. 71.17 ± 6.97 %; Figure 2G-I), macrophages (60.47 ± 3.71 % vs. 57.13 ± 2.75 %; Figure 2J-L) and lymphocytes (22.62 ± 7.24 % vs. 27.26 ± 9.05 %; Figure 2M-O).

### TTA effects on genes related to inflammation and oxidative stress in heart and aorta

**Figure 3:**
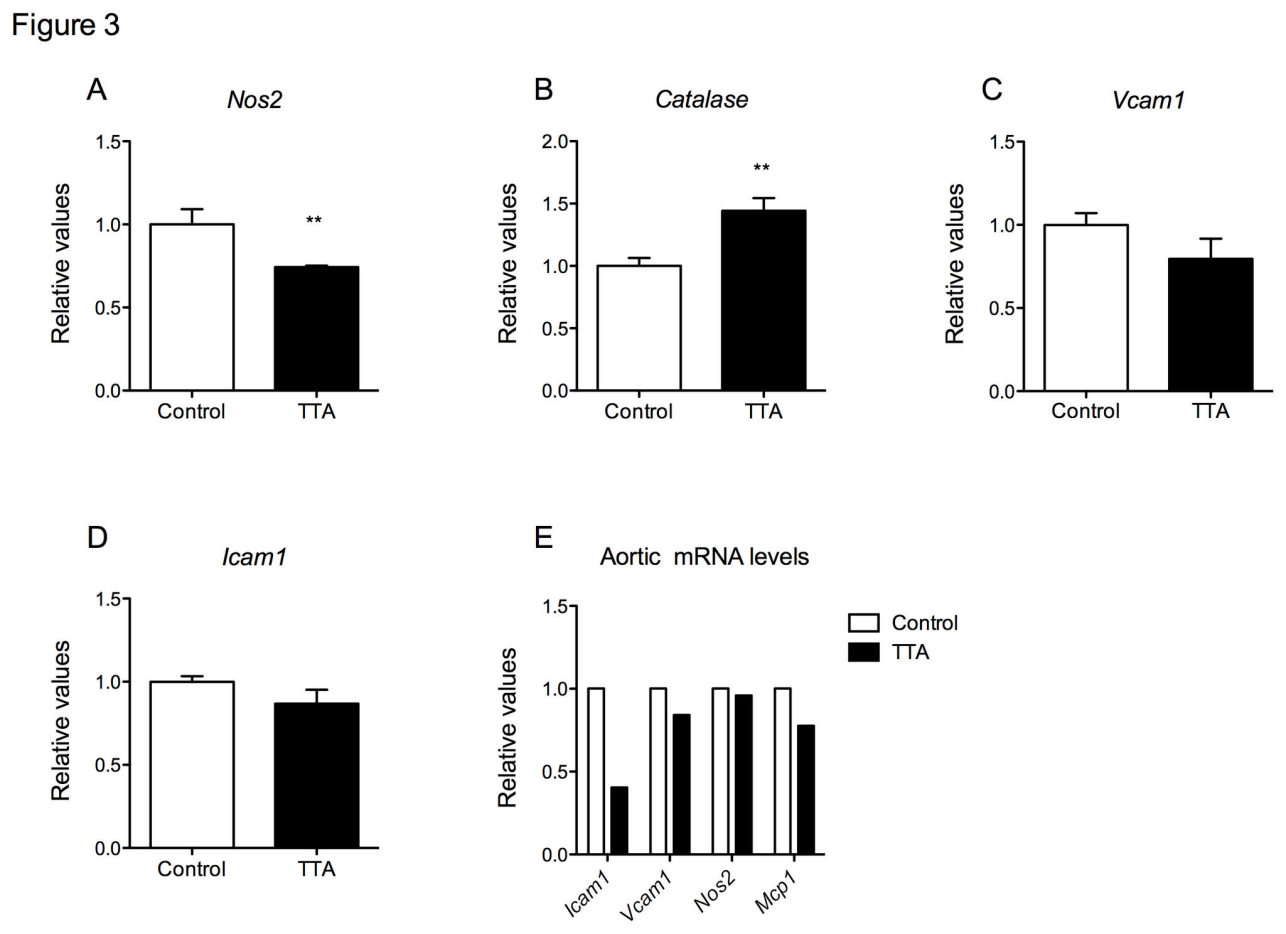
Relative mRNA levels of inflammatory and oxidative stress markers in heart of apoE^-/-^ mice fed a high-fat diet (control) or a diet supplemented with 0.3% TTA after 12 weeks. **A**) Nos2, **B**) Catalase, **C**) *Icam1*, **D**) *Vcam1* and **E**) Aortic arch. Bars represent means ± SD for 3-4 mice for each diet. Mann-Whitney test was used to detect statistical significance and results significantly different from control are indicated **P<0.01.

Enhanced inflammation and oxidative stress seem to be recurring factors in atherogenesis. Therefore it was of interest to observe that the gene expression of inducible nitric oxidase (*Nos2*) was significantly decreased in the heart of TTA-treated apoE^-/-^ mice compared to controls (Figure **3A**), whereas gene expression of the key antioxidant enzyme, catalase (*Cat*) was significantly increased (Figure **3B**). The mRNA expression of *Vcam1* and intracellular adhesion molecule 1 (*Icam1*) in the heart tended to be decreased by TTA administration, but the differences did not reach statistical significance (Figure 3C, D). The gene expression of the inflammatory mediators *Mcp1* and *Tnfa*, and the antioxidants soluble superoxide dismutase (*Sod1*) and mitochondrial superoxide dismutase (*Sod2*) were unchanged (data not shown). The mean mRNA level of *Icam1*, *Vcam1* and *Mcp1* decreased in pooled aortic arch from six animals, whereas mRNA *Nos2* levels seemed to be unchanged by the treatment (Figure **3E**).

### Effect of TTA on systemic inflammation

**Figure 4:**
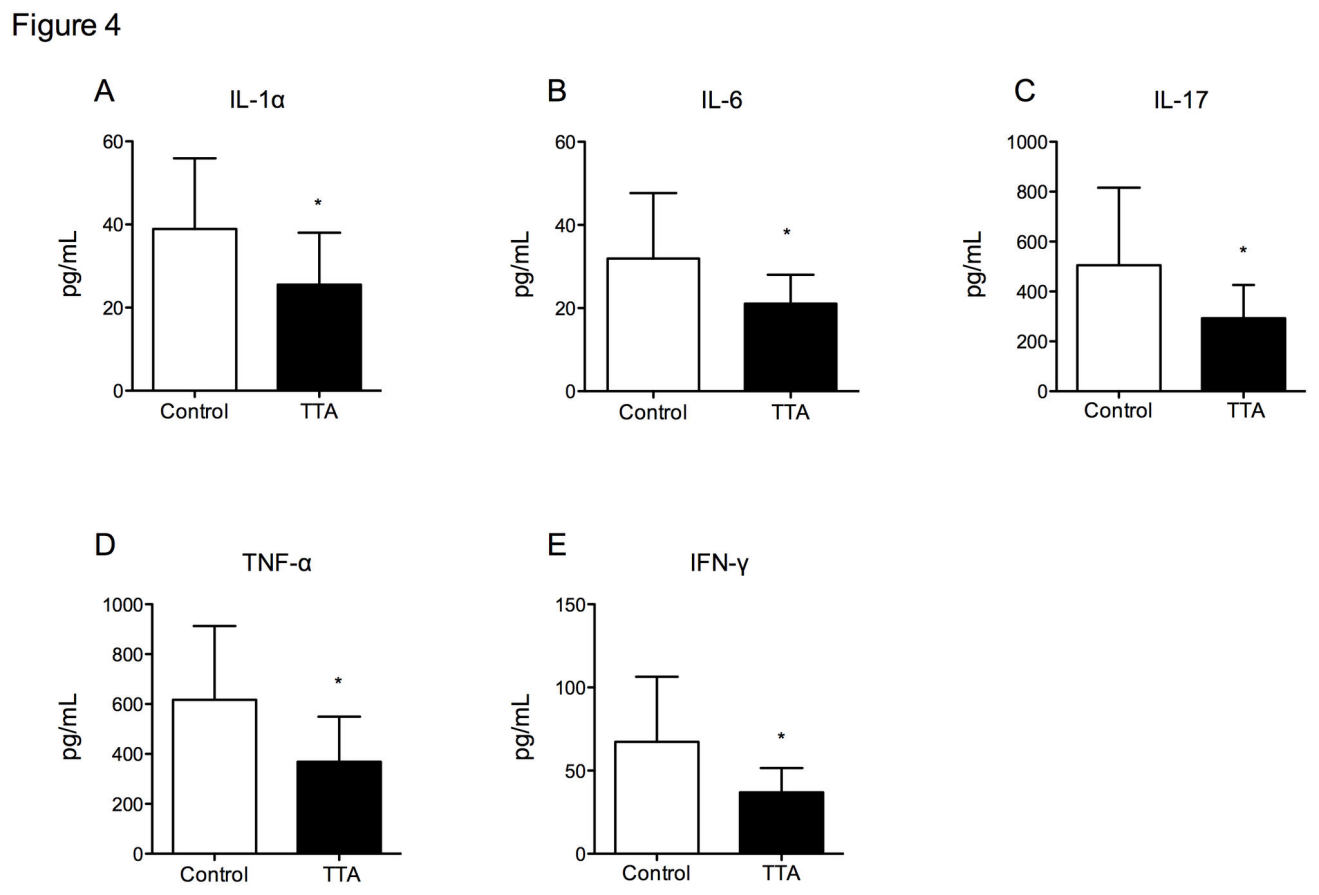
Plasma concentration of inflammatory mediators in apoE^-/-^ mice fed a high-fat diet (control) or a diet supplemented with 0.3% TTA for 11 weeks. Blood samples collected at day 77 of treatment were analysed **A**) IL-1α, **B**) IL-6, **C**) IL-17, **D**) TNF-α and **E**) IFN-γ. Bars represent means ± SD for 4 mice for each diet. Unpaired *t*-test was used to assess statistical significance and results significantly different from control are indicated *P<0.05, **P<0.01, ***P<0.001.

In addition to its potential anti-inflammatory effect within the aortic arch, TTA administration decreased plasma levels of several inflammatory cytokines and chemokines in apoE^-/-^ mice. As shown in Figure **4**, IL-1α, IL-6, IL-17, TNF-α and IFN-γ levels were significantly lower in TTA-treated mice compared to controls. IL-6, IL-17, TNF-α and IFN-γ levels significantly and directly correlated with mouse aortic plaque area (r = 0.70, p=0.011; r = 0.74, p=0.006; r = 0.68, p=0.015; r = 0.64, p=0,027, respectively). No significant changes of IL-1β, IL-10, and MCP-1 could be observed after TTA administration (data not shown).

### Effect of TTA on lipid concentrations in plasma

**Figure 5:**
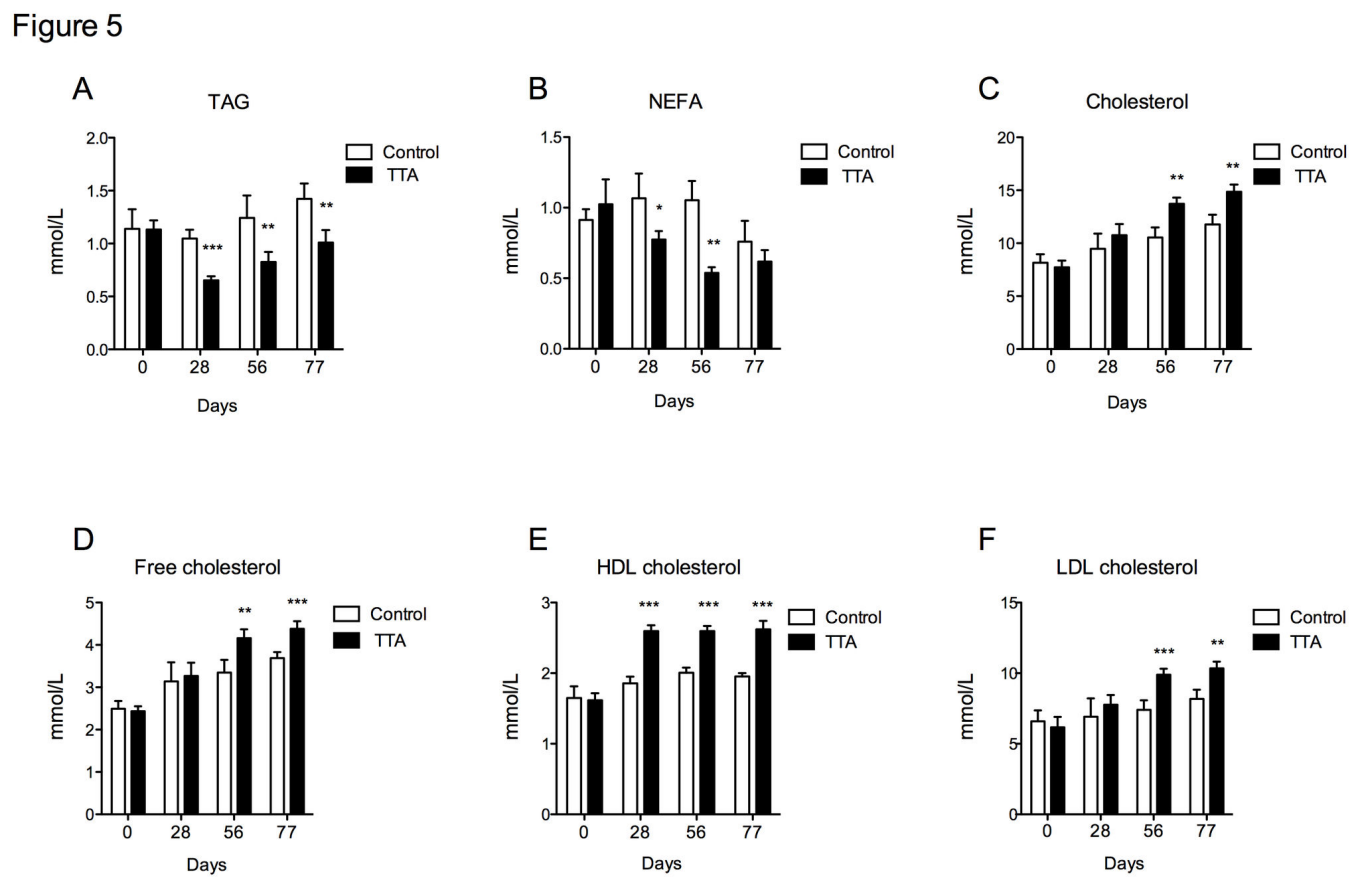
Plasma lipid concentrations of apoE^-/-^ mice fed a high-fat diet (control) or a diet supplemented with 0.3% TTA. After 28, 56 and 77 days of diet induction, blood was collected, EDTA plasma was isolated and plasma lipids were determined by enzymatic assay. **A**) Triacylglycerols, **B**) Non-esterified fatty acids, **C**) Cholesterol, **D**) Free cholesterol, **E**) High-density lipoprotein cholesterol and **F**) Low-density lipoprotein cholesterol. Bars represent means ± SD for 4 mice for each diet. Unpaired *t*-test was used to detect statistical significance and results significantly different from control are indicated *P<0.05, **P<0.01, ***P<0.001.

The plasma concentrations of TAGs and NEFAs were significantly reduced in the TTA-treated mice compared to the control group already after 28 days of treatment (Figure 5A, B). Interestingly, plasma total and free cholesterol levels were increased (Figure 5C, D), as well as levels of HDL- and LDL-cholesterol (Figure 5E, F) whereas the plasma phospholipid concentration and the ratio of HDL/LDL-cholesterol were unaffected by TTA administration compared to the controls (data not shown).

### Effect of TTA on fatty acid composition in heart and plasma

**Figure 6:**
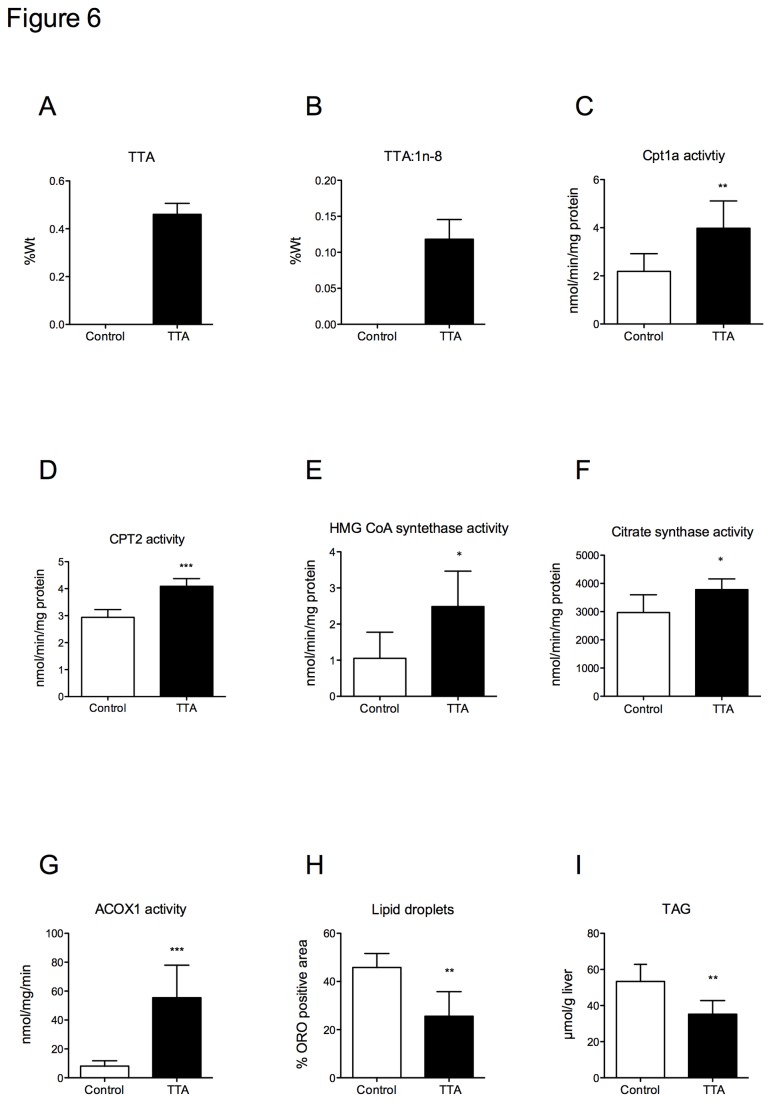
Hepatic paramters in apoE^-/-^ mice fed a high-fat diet (control) or a diet supplemented with 0.3% TTA for 12 weeks. In liver extracts, fatty acids were measured using gas-liquid chromotography, enzyme activities were measured spectrophotometrically and lipid deposition was assessed using Red Oil staining. **A**) Tetradecylthioacetic aicd and **B**) TTA:1n-8, **C**) Carnitine palmitoyltransferase 1a activity, **D**) carnitine palmitoyltransferase 2 **E**) 3-hydroxy-3-methylglutaryl coenzyme A synthase activity **F**) Citrate synthase activity, **G**) Acyl-coenzyme A oxidase activity, **H**) Lipids droplets and **I**) Triacylglycerols. Bars represent means ± SD in 4-6 mice for each diet. Unpaired *t*-test was used to detect statistical significance and results significantly different from control are indicated *P<0.05, **P<0.01, ***P<0.001.

Dietary supplementation with 0.3% TTA promoted different effects on heart and plasma fatty acid composition in apoE^-/-^ mice fed a high-fat diet (Table **1**). While TTA had no effect in the relative amounts of saturated fatty acids (SFA) in heart, it reduced the weight % of SFA in plasma, mainly as a consequence of reduced due amount of stearic acid (C18:0) (Table **1**). In contrast, TTA had no significant effect on the relative amount of monounsaturated fatty acid in heart or plasma (Table **1**). Δ9 desaturated TTA in addition to TTA was found in heart and plasma (Table **1**) demonstrating an activity of SCD1. TTA significantly increased the relative amounts of n-9 polyunsaturated fatty acids (PUFAs) in both heart and plasma, mainly because of increased relative amounts of mead acid (C20:3n-9) (Table **1**). While TTA induced a decrease in n-3 PUFAs in plasma, due to decreased relative amounts of C18:3n-3, C18:4n-3, C20:5n-3 and C22:6n-3, the relative amounts of n-3 PUFAs tended to increase during TTA treatment in the heart, due to significantly increased amounts of EPA (C20:5n-3) and DPA (C22:5 n-3) (Table **1**). TTA decreased the weight % of n-6 PUFAs in heart, mainly reflecting a reduction in the prostaglandin precursor arachidonic acid (20:4n-6). TTA increased n-6 PUFAs in plasma and this was due to increased relative amounts of C20:3n-6, C20:4n-6 and C22:4n-6 (Table **1**). Altogether, TTA administration for 12 weeks influenced the ratio of n-3 to n-6 PUFAs in two different ways: in heart an increase was observed, whereas in plasma a reduction was found (Figure **6H**). 

**Table 1:** Fatty acid composition in heart and plasma of apoE^-/-^ mice fed a high-fat diet (control) or a diet supplemented with 0.3% TTA for 12 weeks.

**Fatty acids**	**Heart**		**Plasma**	
	**Control**	**TTA**	**Control**	**TTA**
**SFAs**	31 ± 0.68	31 ± 0.84	32 ± 0.47	32 ± 0.16
**C18:0**	16 ± 0.59	16 ± 1.11	12 ± 0.20	8.8 ± 0.20***
**C22:0**	0.17 ± 0.01	0.20 ± 0.02	0.40 ± 0.02	0.40 ± 0.03**
**C23:0**	0.05 ± 0.00	0.08 ± 0.01**	0.16 ± 0.02	0.21 ± 0.02**
**C24:0**	0.09 ± 0.01	0.12 ± 0.01*	0.31 ± 0.02	0.36 ± 0.02*
**TTA**	<0.00	32 ± 0.69*	<0.00	33 ± 0.20
**MUFAs**	21 ± 1.9	18 ± 4.4	31 ± 0.42	29 ± 0.81***
**C18:1n-7**	2.0 ± 0.05	2.0 ± 0.04	1.3 ± 0.03	1.2 ± 0.04**
**n9 PUFAs**	0.13 ± 0.00	0.24 ± 0.04*	0.21 ± 0.31	0.41 ± 0.29
**C18:1n-9**	17 ± 1.7	14 ± 3.7	25 ± 0.38	24 ± 0.77*
**C20:3n-9**	0.13 ± 0.00	3.2 ± 0.04*	0.2 ± 0.01	0.4 ± 0.03***
**TTA:1n-8**	<0.00	37 ± 2.4*	<0.00	30 ± 0.81
**n6 PUFAs**	26 ± 0.61	23 ± 1.0*	28 ± 0.37	32 ± 0.82***
**C20:3n-6**	0.66 ± 0.02	1.2 ± 0.18**	0.5 ± 0.01	1.2 ± 0.04***
**C20:4n-6**	8.7 ± 0.37	7.0 ± 0.64*	12 ± 0.4	15 ± 0.9***
**C22:4n-6**	0.77 ± 0.03	0.80 ± 0.11	0.21 ± 0.01	0.28 ± 0.02**
**n3 PUFAs**	23 ± 1.4	26 ± 4.0	6.4 ± 0.01	5.3 ± 0.10***
**C18:3n-3**	0.23 ± 0.01	0.20 ± 0.06	0.60 ± 0.09	0.40 ± 0.02**
**C18:4n-3**	<0.00	<0.00	0.05 ± 0.00	0.01 ± 0.00***
**C20:5n-3**	0.06 ± 0.00	0.11 ± 0.02*	0.5 ± 0.04	0.3 ± 0.00**
**C22:5n-3**	1.7 ± 0.08	3.0 ± 0.57*	0.31 ± 0.02	0.35 ± 0.03*
**C22:6n-3**	21 ± 1.3	23 ± 3.6	5.0 ± 0.3	4.2 ± 0.1**
**n3/n6**	0.88 ± 0.07	1.1 ± 0.12*	0.23 ± 0.01	0.17 ± 0.01*

Values are given as % (w/w) and shown as means ± SD for 6 mice for each diet. *P<0.05, **P<0.01, ***P=0.001.

### Hepatic gene and enzyme activities

TTA and delta 9 desaturated TTA were also found in liver of TTA-treated mice (Figure 6A, B), indicating that TTA-CoA is a suitable substrate for SCD1. The mRNA level of *Scd1*, however, was unchanged by TTA administration (Table **2**). Under normal condition, fatty acids to the liver can either be oxidized or secreted in the VLDL-particles. Gene expression of *Cd36* was up-regulated in TTA-treated mice (Table **2**) indicating higher influx of fatty acids to the liver. We found that the activities of both CPT1 and CPT2 were stimulated in the TTA-fed mice compared to controls (Figure 6C, D, respectively), in addition to increased HMG-CoA synthase activity (Figure **6E**) demonstrating increased mitochondrial fatty acid oxidation and ketogenesis. This was also associated with higher levels of the mitochondrial biomarker citrate synthase (Figure **6F**). Up-regulation of these enzymes, as well as of ACOX1 (Figure **6G**) by TTA administration, supports PPARα activation. The mRNA expression of *Ppara*, however, was unchanged (Table **2**). The lipid droplets and the hepatic level of total TAG were decreased in TTA-treated apoE^-/-^ mice (Figure 6H, I). Hepatic TAG biosynthesis and VLDL secretion seemed to be unaffected by TTA treatment as the gene expression of *Gpat* and *ApoB* were similar in TTA-treated mice compared to controls (Table **2**). The mRNA expression of VLDL receptor (*VLDLr*) was significantly increased (Table **2**). Reduced hepatic TAG content may partly be due to reduced lipogenesis as the mRNA expression of acetyl carboxylase alpha (*Acaca*) was decreased by TTA administration (Table **2**).

**Table 2:** Hepatic gene expression om apoE^-/-^ mice after 12 weeks of dietary supplementation.

**Lipid transport and β-oxidation**		**Diet groups**		**P value**
**Gene**	**Function**	**Control**	**TTA**	
*Apob*	Fatty acid transport	1.00 ± 0.09	0.87 ± 0.17	0.501
*Cd36*	Fatty acid import	1.00 ± 0.20	2.23 ± 0.52*	**0.045**
*Cpt1a*	β-oxidation	1.00 ± 0.27	0.55 ± 0.15	0.252
*Cpt2*	β-oxidation	1.00 ± 0.04	1.18 ± 0.16	0.260
*Vldlr*	Fatty acid import	1.00 ± 0.11	2.46 ± 0.56*	**0.029**
**Lipogenesis, desaturation and transcription**		**Diet groups**		**P value**
**Gene**	**Function**	**Control**	**TTA**	
*Acaca*	Fatty acid synthesis	1.00 ± 0.08	0.676 ± 0.03	0.203
*Gpat*	Glycerolipid synthesis	1.00 ± 0.13	1.44 ± 0.14	0.075
*Scd1*	Desaturation	1.00 ± 0.14	0.72 ± 0.02	0.161
*Ppara*	Energy homeostasis	1.00 ± 0.20	0.68 ± 0.33	0.463

Data are shown as relative values ± SD 4 mice for each diet. *P<0.05.

## Discussion

TTA is reported to be an important regulator of lipids through PPARs [12,16,31] and to have positive effects on plasma lipid risk factors related to cardiovascular diseases [32] and most probably to atherosclerosis [33]. Therefore, the ability of TTA to inhibit the development of atherosclerosis in apoE^-/-^ mice fed on a high-fat diet was investigated. Notably, TTA was found to reduce atherosclerotic plaque area in the aortic arch in these mice. In addition, a decrease in lesion area was observed at the aortic sinus of TTA-treated mice, but failed to be significant possibly due to small sample size. However, our results suggest a potential beneficial effect of TTA treatment on plague formation.

The atherosclerotic process is initiated by accumulation of plasma lipoproteins in the subendothelial space of the blood vessel and uptake of these lipoproteins by machrophages will result in their transformation into foam cells [34]. Noteworthy, the plasma cholesterol, free cholesterol, HDL- and LDL-cholesterol levels were increased by TTA treatment, suggesting that effect of TTA on plaque reduction is not related to its cholesterol lowering properties. Inflammation has emerged as an important factor in the development of atherosclerosis characterized by a state of non-resolving inflammation. In contrast to the lack of effect on plasma cholesterol, TTA induced a marked decrease in plasma levels of several inflammatory mediators with relevance to atherogenesis such as IL-1α, IL-6, IL-17, TNF-α and IFN-γ. Moreover, TTA also induced a decrease in the gene expression of *Icam1*, *Vcam1* and *Mcp1* within the aortic arch, suggesting anti-inflammatory effects of TTA both systemically and within the lesions. Although we have no firm evidence, it is tempting to hypothesize that the atheroprotective effect of TTA in apoE^-/-^ mice involves both anti-inflammatory and anti-oxidative effects of this fatty acid. 

The effects of TTA on fatty acid composition could also be of relevance for the effect of TTA on plaque size. Thus, while TTA treatment resulted in significant increased plasma level of arachidonic acid (C20:4n-6), the precursor for prostaglandins [35], and a tendency to reduced amount of the anti-inflammatory n-3 PUFAs in plasma, TTA increased the amount of the n-3 PUFAs EPA (C20:5n-3) and DHA (C22:6n-3), and decreased the amount C20:4n-6 within the heart. We have previously shown that TTA is taken up in endothelial cells [16] and macrophages [36], and after local delivery reduces the local inflammatory responses and collagen accumulation [37]. TTA inhibits proliferation of smooth muscle cells and has the ability to attenuate TNFα-mediated endothelial cell activity involving both PPARα-dependent and –independent pathways [16]. As previously reported, TTA was taken up in the arterial wall [18]. Thus, its anti-atherosclerotic effect could be a direct action on the vascular cells with anti-inflammatory and antioxidative effects, at least partly related to its effect of the local fatty acid composition.

In normolipidemic rats [38] and chronic inflammatory mice [39] the plasma levels of TAGs and NEFAs, as well as the hepatic concentration of TAGs, are regulated by the hepatic mitochondrial activity after TTA treatment. In the present study, the increased mRNA level of *Cd36*, which may be involved in fatty acid translocase, could result in an increased fatty acid uptake by the liver. The activation of fatty acids to their CoA -esters, catalyzed by long-chain acyl-CoA synthetase, is probably stimulated by TTA administration [39]. Plasma TAG levels depend on the balance between TAG synthesis, degradation and catabolism by the liver, as well as on its intravascular catabolism. We have previously reported that thia fatty acid treatment resulted in a more pronounced decrease in plasma TAGs than the decrease VLDL-secretion, indicating that changes in clearance of VLDL might contribute to the reduction in plasma TAGs [27]. Indeed, the lipoprotein lipase (LPL) mRNA in 3T3-L1 preadipocytes was increased by TTA [40]. Thus, this LPL action may well result in a decrease of plasma TAGs in TTA-treated apoE^(-/-)^ mice. The apoE^-/-^ mice exhibited increased hepatic activities of both CPT1 and CPT2 and a slightly up-regulation of CPT2 could be confirmed at the mRNA level. Moreover, proliferation of mitochondria and peroxisomes seemed to take place in the liver of apoE^-/-^ mice as activities of both citrate synthase and ACOX1 were increased by TTA supplementation. TTA significantly reduced the hepatic TAG level in apoE^-/-^ mice and this is most probably due to increased fatty acid catabolism and decreased lipogenesis as the gene expression of *Acaca* was reduced. Both TTA-treated mice and control mice displayed similar weight gain during the study, showing that our beneficial effects of TTA are not simply due to suppression of appetite, lack of palatability or blockage of intestinal uptake of dietary fatty acids. Overall, we hypothesize that the triggered TAG metabolism accompanied with increased mitochondrial function in liver may contribute to the beneficial action of TTA in apoE^-/-^ mice.

The present study has some limitations such as the lack of protein data and lack of data on fatty acid composition within the lesion. However, our observations suggest that the modified fatty acid analogue, TTA, inhibits atherosclerosis development *in vivo*, an effect most likely independent of changes in plasma cholesterol levels, but related to an inflammatory response both at vascular and systemic levels, in addition to an antioxidant effect at the vascular level. This could be related to the effect of TTA on local fatty acid composition as well as its effect on mitochondrial function in the liver. Forthcoming studies should further explore the mechanism by which TTA exerts its anti-atherosclerotic effects. If its athero-protective effects are confirmed in additional studies on experimental atherosclerosis, TTA should be explored as treatment modality in clinical atherosclerosis. 

## Supporting Information

Table S1 **Composition of the diets shown as g/kg diet.** The diets were isocaloric and isonitrogenous.
(DOCX)

Table S2 **Overview of analyzed genes.**
(DOCX)
