# Supplementary material for: An Immunomodulating Fatty Acid Analogue Targeting Mitochondria Exerts Anti-Atherosclerotic Effect beyond Plasma Cholesterol-Lowering Activity in apoE-/- Mice
Source: PLoS One. 2013 Dec 4;8(12):e81963. doi: 10.1371/journal.pone.0081963 (PMC3852987; doi:10.1371/journal.pone.0081963)
Supplement: Table S1 — Composition of the diets shown as g/kg diet. The diets were isocaloric and isonitrogenous. (DOCX) [file pone.0081963.s001.docx]

**Table S1. Diet composition**

| **Ingredients** | **Control** | **TTA** |
| --- | --- | --- |
| ***Protein source*** |  |  |
| **Casein** | 250 | 250 |
| ***Fat source*** |  |  |
| **Soy oil** | 24 | 24 |
| **Lard** | 213 | 210 |
| **TTA** |  | 3.0 |
| ***Carbohydrates*** |  |  |
| **Cornstarch** | 105 | 105 |
| **Dyetrose** | 154 | 154 |
| **Sucrose** | 117 | 117 |
| **Fiber** | 58 | 58 |
| ***Micronutrients*** |  |  |
| **AIN-93G-MX mineral mix** | 41 | 41 |
| **AIN-93-VX vitamin mix** | 12 | 12 |
| **L-Cysteine** | 3.5 | 3.5 |
| **Choline bitartrate** | 2.9 | 2.9 |
| **tert-Butyl-hydroquinone** | 0.016 | 0.016 |
| **Dextrin/Cellulose** | 20 | 16 |

Amounts shown are g/Kg diet. The diets are isocaloric and isonitrogenous.
