# Supplementary material for: An Immunomodulating Fatty Acid Analogue Targeting Mitochondria Exerts Anti-Atherosclerotic Effect beyond Plasma Cholesterol-Lowering Activity in apoE-/- Mice
Source: PLoS One. 2013 Dec 4;8(12):e81963. doi: 10.1371/journal.pone.0081963 (PMC3852987; doi:10.1371/journal.pone.0081963)
Supplement: Table S2 — Overview of analyzed genes. (DOCX) [file pone.0081963.s002.docx]

**Table S2:** Gene names of analysed genes, primer/probe sets, and analysed tissues

| **Gene** | **Full name** | **AoD no.^1^** | **Liver** | **Heart** | **Aorta** |
| --- | --- | --- | --- | --- | --- |
|  |  |  |  |  |  |
| ***Acaca*** | Acetyl-CoA carboxylase alpha | Mm01304277_m1 | x |  |  |
| ***Acox1*** | Acyl-CoA oxidase 1 | Mm0043579 | x |  |  |
| ***Apob*** | Apolipoprotein B | Mm01545156 | x |  |  |
| ***Cpt1a*** | Carnitine palmitoyltransferase 1 | Mm00550438 | x |  |  |
| ***Cpt2*** | Carnitine palmiotyltransferase 2 | Mm00487202 | x |  |  |
| ***Cd36* (*Fat*)** | CD36 antigen | Mm00432403 | x |  |  |
| ***Gpat*** | Glycerol-3-phosphate acyltransferase | Mm00833328_m1 | x |  |  |
| ***Ppara*** | Peroxisome proliferator-activated receptor alpha |  |  |  |  |
| ***Scd1*** | Stearoyl-CoA desaturase 1 | Mm00772290_m1 | x |  |  |
| ***Vldlr*** | Very low-density lipoprotein receptor | Mm00443281 | x |  |  |
| ***Cat*** | Catalase | Mm00437992_m1 |  | x |  |
| ***Mcp1*** | Monocyte chemoattractant protein | Mm00441242 |  | x | x |
| ***Icam1*** | Intracellular adhesion molecule | Mm00516023_m1 |  | x | x |
| ***Nos2*** | Nitric oxide synthase 2 | Mm00440502_m1 |  | x | x |
| ***Sod1*** | Superoxide dismutase 1, soluble | Mm01344233_g1 |  | x |  |
| ***Sod2*** | Superoxide dismutase 2, mitochondrial | Mm01313000_m1 |  | x |  |
| ***Tnfa*** | Tumor necrosis factor alpha | Mm00443260_g1 |  | x |  |
| ***Vcam1*** | Vascular cell adhesion molecule 1 | Mm00443281 |  | x | x |

^1^ Catalogue number of custom TaqMan gene expression primer/probe sets from Applied Biosystems.
